# Thermoanalytical and Kinetic Studies for the Thermal Stability of Emerging Pharmaceutical Pollutants Under Different Heating Rates

**DOI:** 10.3390/jox14040095

**Published:** 2024-11-14

**Authors:** Christian Ebere Enyoh, Tochukwu Oluwatosin Maduka, Miho Suzuki, Senlin Lu, Qingyue Wang

**Affiliations:** 1 Graduate School of Science and Engineering, Saitama University, 255 Shimo-Okubo, Sakura-ku, Saitama City 338-8570, Japan; maduka.t.o.205@ms.saitama-u.ac.jp (T.O.M.); miho@mail.saitama-u.ac.jp (M.S.); 2 School of Environmental and Chemical Engineering, Shanghai University, Shanghai 200444, China; senlinlv@staff.shu.edu.cn

**Keywords:** degradation kinetics, thermogravimetric analysis (TGA), differential thermal analysis (DTA), isoconversional kinetics, waste treatment, thermodynamic parameters

## Abstract

Emerging pharmaceutical pollutants like ciprofloxacin (CIP) and ibuprofen (IBU) are frequently detected in aquatic environments, posing risks to ecosystems and human health. Since pollutants rarely exist alone in the environment, understanding the thermal stability and degradation kinetics of these compounds, especially in mixtures, is crucial for developing effective removal strategies. This study therefore investigates the thermal stability and degradation kinetics of CIP and IBU, under different heating rates. Thermogravimetric analysis (TGA) and differential thermal analysis (DTA) were employed to examine the thermal behavior of these compounds individually and in mixture (CIP + IBU) at heating rates of 10, 20, and 30 °C/min. The kinetics of thermal degradation were analyzed using both model-fitting (Coats–Redfern (CR)) and model-free (Kissinger–Akahira–Sunose (KAS), Flynn–Wall–Ozawa (FWO), and Friedman (FR)) methods. The results showed distinct degradation patterns, with CIP decomposing between 280 and 550 °C and IBU between 152 and 350 °C, while the mixture exhibited multistep decomposition in the 157–500 °C range. The CR model indicated first-order kinetics as a better fit for the degradation (except for IBU). Furthermore, CIP exhibits higher thermal stability and activation energy compared to IBU, with the KAS model yielding activation energies of 58.09 kJ/mol for CIP, 11.37 kJ/mol for IBU, and 41.09 kJ/mol for CIP + IBU mixture. The CIP + IBU mixture generally showed intermediate thermal properties, suggesting synergistic and antagonistic interactions between the compounds. Thermodynamic parameters (Δ*H*°, Δ*G*°, Δ*S*°) were calculated, revealing non-spontaneous, endothermic processes for all samples (except in the FWO method) with a decrease in molecular disorder and positive Δ*G*° values across all models and heating rates. The study found that higher heating rates led to less thermodynamically favorable conditions for degradation. These findings provide important information concerning the thermal behavior of these pharmaceutical pollutants, which can inform strategies for their removal from the environment and the development of more effective waste-treatment processes.

## 1. Introduction

Pharmaceutical pollutants in the environment present an emerging concern due to their potential harmful effects on ecosystems and human health [1]. Among the most notable of these contaminants are ciprofloxacin (CIP) and ibuprofen (IBU), both of which are extensively used and exhibit environmental persistence. A common synthetic fluoroquinolone antibiotic substance used to treat bacterial infections is called CIP [2]. Nonetheless, due to its extensive usage, CIP is frequently found in a range of aquatic environments and is released into the environment via wastewater discharges [2,3]. IBU is a non-steroidal anti-inflammatory medication (NSAID) that is used to treat inflammation, fever, and muscular soreness [4]. It is still one of the most often given NSAIDs nowadays [5]. The environmental release of untreated or insufficiently treated wastewater, along with the extensive use of CIP and IBU in veterinary and human medicine, has led to their recognition as emerging pollutants. These pollutants have been detected in soil, groundwater, and surface rivers [4]. Their persistence makes them difficult to degrade and contributes to their accumulation in water bodies and soil, posing risks to aquatic organisms and potentially leading to the development of antibiotic-resistant bacteria [3,6,7,8]. Given the widespread presence of these pharmaceuticals in the environment, it is critical to understand their degradation mechanisms in order to devise effective mitigation strategies.

Thermal analysis techniques have emerged as valuable tools for studying the degradation kinetics and thermodynamic behavior of pharmaceutical compounds [9,10]. By applying these methods, researchers can gain insights into the stability and breakdown processes of pharmaceuticals under varying conditions [11]. A popular method for examining the thermal degradation of pharmaceuticals is non-isothermal thermogravimetric analysis (TGA), which measures mass changes while the substance is heated steadily [9,10]. Researchers can obtain kinetic parameters using either model-fitting or isoconversional approaches thanks to this method’s provision of crucial data for kinetic analysis [11]. However, because model-fitting involves inherent errors in kinetic parameter determination, isoconversional methods are chosen among the different methodologies to analyze non-isothermal solid-state reaction kinetics as recommended by the Kinetics Committee of the International Confederation for Thermal Analysis and Calorimetry (ICTAC) [12]. Isoconversional methods do not require assumptions about the reaction model, enabling the evaluation of effective activation energies without restricting the analysis to a single-step reaction process [12].

There is a notable scarcity of studies focusing on the thermal degradation CIP and ibuprofen IBU singly and mixed in the literature. Tita et al. [13] explored the thermal breakdown of IBU using various isoconversional methods, including Chang, Freeman–Carroll, Friedman (FR), Kissinger–Akahira–Sunrose (KAS), and Flynn–Wall–Ozawa (FWO). Ramukutty and Ramachandran [9] further examined the decomposition kinetics of racemic IBU crystals through non-isothermal analysis, revealing that IBU is thermally stable up to 152.6 °C, with initial mass loss attributed solely to evaporation. Using the Coats–Redfern (CR) method, they calculated thermodynamic parameters such as activation energy, pre-exponential factor, entropy, and Gibbs free energy, providing foundational data on IBU’s thermal stability. However, more research is needed to fully understand the degradation of both CIP and IBU, particularly in mixtures, as pharmaceutical pollutants often exist in combination in the environment [14].

Understanding the thermal degradation mechanisms of these compounds requires examining effective activation energies across different conversion degrees to reveal reaction complexity. In addition to kinetics, thermodynamic parameters—enthalpy (Δ*H*°), entropy (Δ*S*°), and Gibbs free energy (Δ*G*°)—are essential to evaluating energy changes and the spontaneity of the degradation process, which are critical for assessing the viability of thermal degradation, especially for energy-production applications [11,13].

However, since pollutants rarely exist alone in the environment [15,16], it is crucial to understand the thermal stability and degradation kinetics of these compounds, especially in mixtures. Interactions between different pollutants can alter degradation pathways, activation energies, and overall stability, affecting how these compounds behave under thermal treatment. Studying these factors not only provides information concerning the complexity of real-world degradation processes but also helps optimize strategies for removing pollutants, ultimately promoting more efficient and sustainable waste management. By accounting for these interactions, researchers can develop more accurate models and effective treatment systems tailored to mixed pollutant scenarios.

In this study, we aim to elucidate the degradation mechanisms of CIP, IBU, and CIP + IBU mixtures using isoconversional thermal analysis at three different heating rates (10, 20, and 30 °C/min). By analyzing the degradation profiles of these compounds using both model-fitting such as Coats–Redfern model and model-free techniques such as Kissinger–Akahira–Sunose (KAS), Flynn–Ozawa–Wall (OFW), and Friedman (FR) model, we can determine the activation energy, reaction order, and other kinetic parameters, which are essential for understanding their behavior in the environment. Furthermore, this study aims to provide valuable insights into the degradation thermodynamics of these emerging pharmaceutical pollutants, contributing to the development of effective strategies for their mitigation and remediation.

## 2. Materials and Methods

### 2.1. Sources of Ciprofloxacin and Ibuprofen

**Figure 1:**
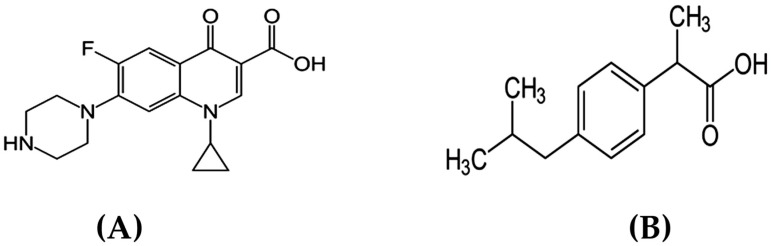
Structure of (**A**) ciprofloxacin and (**B**) ibuprofen. Structure drawn with Chemdraw 8.0.

Ciprofloxacin (CIP: C_17_H_18_FN_3_O_3_, analytical grade, 98%) and ibuprofen (IBU: C_13_H_18_O_2_, analytical grade, 98%) were obtained from Wako Pure Chemical Industries, Japan. The chemicals were used as obtained from the manufacturer without any further purification. The chemical structures of both compounds are shown in Figure 1A,B, respectively.

### 2.2. Thermal Degradation Process

**Figure 2:**
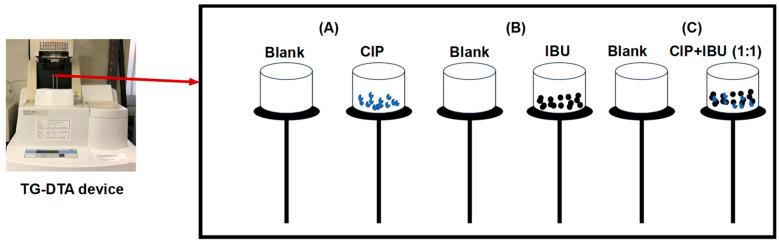
Thermogravimetric analysis of pharmaceutical samples. The study design involved analyzing (**A**) only ciprofloxacin (CIP), (**B**) only ibuprofen (IBU), and (**C**) physical mixture of CIP and IBU at a ratio of 1:1. The left figure is the thermogravimetric analyzer used in this study.

Thermogravimetric analysis (TGA) was conducted to evaluate the degradation process and thermal stability of the pharmaceutical samples, CIP and IBU. The analysis was performed using a TG-DTA device (DTG-60, Shimadzu Corporation). For each sample, 5 mg was placed in a platinum (Pt) cell inside the furnace (Figure 2). Prior to heating, the furnace atmosphere was purged with argon (Ar) gas for 15 min to create an inert environment. The temperature was then increased from room temperature to 700 °C for CIP and CIP + IBU and 350 °C for IBU at varying heating rates of 10, 20, and 30 °C/min, with an Ar flow rate of 100 mL/min. Throughout the heating process, the mass loss of the samples was continuously monitored, and derivative thermogravimetric (DTG) curves were plotted for further analysis.

### 2.3. Kinetic Theory of Breakdown Reaction

Thermogravimetric analysis is used to study the thermal decomposition of solid materials, and the variations in conversion rate with time and temperature under various thermal conditions can provide information about the degradation process, such as the decomposition temperature, the peak temperature, and the average reaction rate [17]. The mathematical formula for the thermal deterioration of materials in non-isothermal conditions is

(1) $$r=\frac{\partial \alpha }{\partial t}=Ae^{(-\frac{E}{RT}){\left(1-\alpha \right)}^{n}}$$

where α is (degree of) conversion, *t* is time, *A* is the pre-exponential factor, *E* is the activation energy, *R* is the universal gas constant (8.314 × 10^−3^ kJ/mol × K), *T* is temperature, and *n* is the reaction order. Conversion can be calculated as follows:

(2) $$\alpha =\frac{m_{o}-m}{m_{o}-m_{f}}$$

where *m_o_* is the sample mass at time = 0; *m* is the sample mass at any specific time/temperature and *m_f_* is the sample mass at the end of TGA experiment.

Some models generated from Equation (1) may be used to obtain kinetic triple parameters from TGA data. The published models either make use of many TGAs at various heating rates (known as isoconversional or model-free techniques) or only one TGA datum (called non-isoconversional or model-fitting methods). In this study, model-fitting techniques such as Coats–Redfern model [18,19], and model-free techniques such as Kissinger–Akahira–Sunose (KAS) [20,21], Flynn–Ozawa–Wall (OFW) [22,23], and Friedman (FR) model [24], were utilized to obtain values for the kinetic parameters and compared.

#### 2.3.1. Non-Isoconversional (Model-Fitting) Analysis

##### Coats–Redfern Method

The present investigation employs the Coats–Redfern (CR) approach, a comprehensive model-fitting technique that entails the fitting of several reaction models to the extent of reaction–temperature curves while concurrently ascertaining the activation energy and frequency factor [18]. This approach, which is shown in Equation (4), approximates the exponential integral using the asymptotic series expansion. For *g*(*α*), however, there are a number of reaction model approximations available in the literature [25]. The authors of this study, nevertheless, chose to use the zero- and first-order rate kinetic model. The first-order rate-determining mechanism postulates that a single reaction route predominates and that the reaction rate is temperature-dependent, whereas the zero-order one proposes that the rate of decomposition is independent of the reacting substance’s concentration. In this case, (4) and (5) approximate zero- and first-order *g*(*α*), and substituting in (3) results in Equations (6) and (7).

(3) $$In\left(\frac{g(\alpha )}{T^{2}}\right)=In\left[\frac{AR}{\beta E}\left(1-\frac{2R\bar{T}}{E}\right)\right]-\frac{E}{RT}$$

*g*(*α*) = *α* (4)

*g*(*α*) = −*ln*(1 − *α*) (5)

(6) $$In\left(\frac{\alpha }{T^{2}}\right)=In\left[\frac{AR}{\beta E}\left(1-\frac{2R\bar{T}}{E}\right)\right]-\frac{E}{RT}$$

(7) $$In\left(-\frac{In(1-\alpha )}{T^{2}}\right)=In\left[\frac{AR}{\beta E}\left(1-\frac{2R\bar{T}}{E}\right)\right]-\frac{E}{RT}$$

The plot $In\left(\frac{\alpha }{T^{2}}\right)$ and $In\left(-\frac{In(1-\alpha )}{T^{2}}\right)$ against 1/*T* gives the intercept and slope of a linear equation and thereafter the values of the activation energy (*E*) and pre-exponential factor (*A*) are calculated from the slope and intercept values.

#### 2.3.2. Isoconversional (Model-Free) Analysis

The isoconversional kinetic models allow for the determination of activation energy (*E*) as a function of the extent of conversion (*α*) without assuming a predefined reaction model. This is particularly important when studying complex systems like pharmaceutical pollutants, where the reaction mechanism may not be straightforward or could vary at different stages of degradation. The application of these models enables the extraction of more reliable kinetic data from non-isothermal experiments, which is critical when assessing the thermal stability and decomposition pathways of environmentally persistent compounds.

##### Kissinger–Akahira–Sunose (KAS) Method

A model-free technique for analyzing the kinetics of thermal material deterioration is the Kissinger–Akahira–Sunose (KAS) approach [26]. It makes it possible to calculate the activation energy (*E*) without making any assumptions about the reaction model. The Arrhenius formulation (Equation (1)) for a particular degree of conversion is where the KAS approach originates. A linear function may be obtained by graphing the natural logarithm of the heating rate divided by the square of the absolute temperature versus the reciprocal of the absolute temperature. To put it another way, this linear function’s slope is exactly proportional to the *E*. This method is based on the expression

(8) $$In\left(\frac{\beta }{T^{2}}\right)=In\left(\frac{AR}{E}\right)-\frac{E_{\alpha }}{RT}$$

This is classified as an isoconversional method as we plot $In\left(\frac{\beta }{T^{2}}\right)$ vs. 1/*T* for degree of conversion, *α*. Using this expression, one obtains the local activation energy *E* at a particular value.

##### Flynn–Wall–Ozawa (FWO) Method

To find the *E* value of a chemical process using thermal analysis, the Flynn–Wall–Ozawa (FWO) technique is a popular integral model-free method that usually uses TGA data. The FWO approach is more flexible for analyzing thermal breakdown reactions than model-fitting techniques since it does not presuppose a particular reaction mechanism [26]. The FWO model evaluates how the activation energy varies with conversion to offer a comprehensive insight of the deterioration behaviour. Studying mixed systems like CIP + IBU, whose component interactions can greatly affect the reaction kinetics, benefits greatly from the model’s independence from reaction order. This method involves the measurement of the temperature *T*, corresponding to a fixed value of degree of conversion, *α*, from the experiments at different heating rates. The FWO method is based on the following equation:

(9) $$Log\;\left(\beta \right)=Log\;\left(\frac{AE}{R}\right)-2.315-0.4567\;\frac{E}{RT}$$

Rearranging Equation (9)

(10) $$Log\;\left(\beta \right)=C-0.4567\;\frac{E}{RT}$$

where *C* = $Log\;\left(\frac{AE}{R}\right)-2.315$ is a constant, which is also equal to the intercept of the linear plot.

The plot of $Log\;\left(\beta \right)$ vs. 1/*T* gives at a specific degree of conversion (in this case *α* = 0.8), the slope − 0.4567*E/*R*, from which the activation energy is evaluated while *A* is further computed from C and the intercept of the linear plot.

##### Friedman (FR) Method

The Friedman (FR) method is a differential isoconversional approach used to calculate the activation energy (*E_a_*) from TGA of the pharmaceutical samples under different heating rates. Unlike integral methods like Flynn–Wall–Ozawa (FWO) or Kissinger–Akahira–Sunose (KAS), the Friedman method is based on the differential form of the Arrhenius equation (Equation (1)), making it more sensitive to small changes in data. This model allows a highly thorough and localized examination of the deterioration process by concentrating on specific conversion intervals. The Friedman model helps identify important points where the response mechanism may alter in this study by highlighting changes in degradation behaviour at various phases of the pollutants’ thermal disintegration. The equation is presented in Equation (11).

(11) $$In\left(\frac{\partial_{\alpha }}{\partial_{t}}\right)=In\left(\beta \frac{\partial_{\alpha }}{\partial_{t}}\right)=In\;(Af\left(\alpha \right))-\frac{E}{RT}$$

Thus, at a constant degree of conversion (*α*), the plot of $In\left(\beta \frac{\partial_{\alpha }}{\partial_{t}}\right)$ vs. 1/*T* obtained from curves recorded at several heating will yield a straight line, and the slope of this line is −*E*/*R*, from which the activation energy can be obtained. Furthermore from $In\;A+In\;f\left(\alpha \right)$, the *A* for first order [$f\left(\alpha \right)=1-\alpha$] and second order [$f\left(\alpha \right)={\left(1-\alpha \right)}^{2}$] can be computed from the intercept of the linear plot.

### 2.4. Thermodynamic Theory of Breakdown Reaction

A thermodynamic analysis is necessary to comprehend any chemical process’s overall performance. We can determine the reasons behind the losses brought on by irreversibility thanks to this analysis. We use the kinetic parameters (obtained from the model-free kinetics) to calculate the thermodynamic parameters in this study of the thermal degradation process of the various pharmaceutical samples: changes in enthalpy (∆*H*°, kJ/mol), entropy (Δ*S*°, kJ/mol × K), and Gibbs free energy (∆*G*°, kJ/mol). Formulas (12)–(14) are used to calculate the values of ∆*H*, ∆*G*, and ∆*S* in order to carry out the thermodynamic analysis. According to [17], these thermodynamic parameters are essential for energy monitoring as well as evaluating the viability and advancement of the thermal degradation process.

(12) ∆H=E−RT

(13) $$∆G=E+RT\ln\left(\frac{k_{b}T}{h\;A}\right)$$

(14) $$∆S=\frac{∆H-∆G}{T}$$

where *T* is temperature in Kelvin (K); *k*_*B*_ is the Boltzmann constant with a value of 1.381 × 10^−23^ J/K; and ℎ is the Planck constant with a value of 6.626 × 10^−34^ J × s.

## 3. Results

### 3.1. Thermal Degradation Pattern at Different Heating Rates

**Figure 3:**
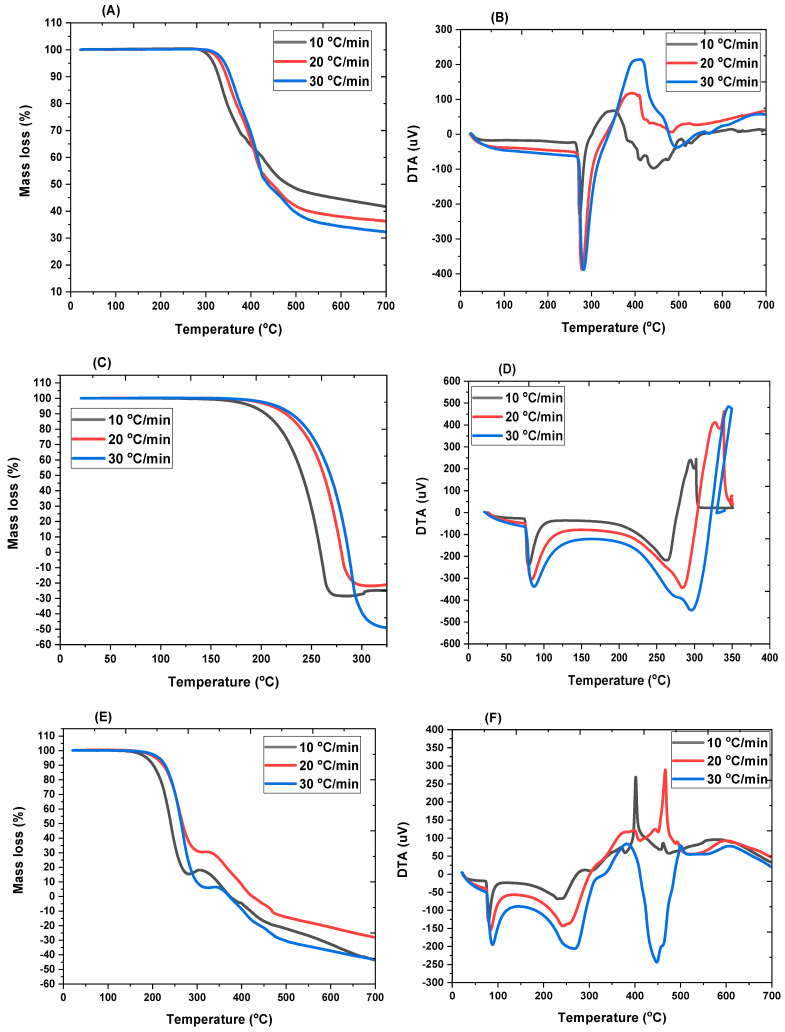
Thermogram of TGA and DTA curves for CIP (**A**,**B**), IBU (**C**,**D**) and CIP + IBU mixture (**E**,**F**).

**Figure 4:**
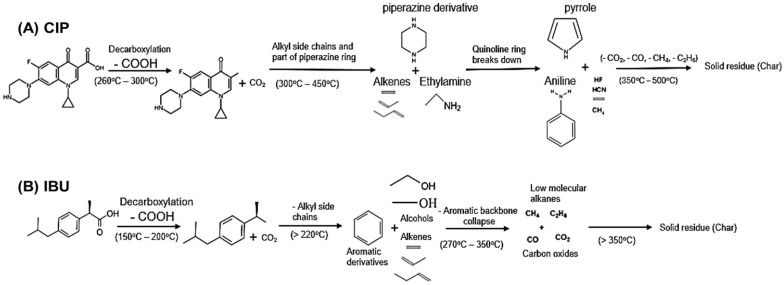
Proposed theoretical pathways for the thermal breakdown mechanism for CIP (**A**) and IBU (**B**) [29,30,31].

TG and DTA curves of the analyzed pharmaceutical samples are shown in Figure 3, while the thermal analysis data showing the onset and endset temperature are summarized in Table 1. We also proposed a theoretical pathway for the thermal decomposition of the sample in Figure 4. In the CIP TGA profile (Figure 3A), no mass loss was observed from room temperature until around 280–300 °C; this may be due the absence of absorbed moisture or water of hydration (25 °C to 280 °C). The sample decomposition occurred between 280 and 550 °C. The first decomposition was observed from temperature of about 280 °C to 420 °C, where about 40% of the sample decomposed. During this stage, it was observed that the sample began decomposing with increasing heating rate; i.e., 10 °C/min heating condition decomposed ahead of the 20°C/min, and 30°C/min heating condition, respectively. This significant mass loss that occurs at this temperature range is indicative of decarboxylation of the carboxyl group, leading to the formation of carbon dioxide (CO_2_) and other volatile byproducts, and further degradation from around 300 °C–420 °C may correspond to the degradation of the alkyl side chains and part of the piperazine ring, leading to the formation of alkenes and other degradation products (Figure 4A). After this temperature, the sample began to decompose in the reverse direction, showing increasing mass loss as the heating rate was increased. This observation occurred around 350 °C and 450 °C, which may be due to the breakage of the quinoline rings, resulting in the formation of hydrogen fluoride, hydrogen cyanide, ethene, methane, pyrrole, and aniline and other degradation products (Figure 4A). This observation continued until the final decomposition stage. At this stage, the sample degraded following higher heating rate as more mass loss was observed at 30 °C/min, followed by 20 °C/min and then 10 °C/min. Here, the degradation is almost complete, and the remaining mass may represent residual inorganic ash or non-combustible components along with the degradation products such as the oxides of carbon and lower molecular alkanes (Figure 4A). To further explain the degradation process, the DTA curve was also examined (Figure 3B). The DTA curve observed a sharp endothermic peak around 260 °C–300 °C indicating the melting of CIP and decarboxylation [27]. Exothermic peaks observed between 300 °C and 450 °C, depending on the heating rate, indicate the breakdown of ciprofloxacin’s (CIP) molecular structure. This temperature range marks the critical point of thermal stability, where CIP begins to degrade and lose its pharmacological effectiveness. Around 350 °C–500 °C, the final decomposition appeared in the DTA curves. These endothermic peaks show the final stages of the breakdown of organic matter, resulting in the formation of carbon dioxide (CO_2_) and other gaseous products. It also coincides with major mass loss in TGA. The probable mechanism is presented in Figure 4A.

In the TGA of IBU (Figure 3C), two decomposition stages were observed between 152 °C and 350 °C, which is in agreement with previous studies [9,13]. Decarboxylation occurred between 152 °C and 200 °C, releasing carbon dioxide (CO_2_). The decomposition at 152 °C, 190 °C, and 210 °C (under heating rates of 10, 20, and 30 °C/min, respectively) likely involved the volatilization of low-molecular-mass hydrocarbons (Figure 4B). Above 220 °C, alkyl chain breakdown produced aromatic derivatives, lower molecular mass alcohols, and alkenes. From 270 °C to 350 °C (Figure 3C), the aromatic backbone collapsed, resulting in the release of carbon oxides and lower molecular mass alkanes (Figure 4B). Beyond 350 °C, further degradation may lead to the formation of residual ash and carbon, marking the final stage of decomposition (Figure 4B). The decomposition profile showed a clear dependence on heating conditions, with higher heating rates accelerating mass loss. Information gathered from the DTA curves showed that at around 75 °C–80 °C a sharp endothermic peak in the DTA curve, which indicated the melting of the IBU sample; IBU is known to melt in this temperature range [28]. The energy absorbed may correspond to the phase transition from solid to liquid (Figure 3D). From 200 °C to 300 °C, ibuprofen undergoes significant thermal degradation, beginning with the breakdown of its functional groups. The large, pronounced endothermic peaks observed from each DTA curve in this temperature range indicate thermal decomposition of IBU. As IBU degrades further, any remaining carbonaceous material may react with oxygen, releasing heat (an exothermic process); this can be explained by the slight endothermic peaks observed between 300 °C and 350 °C (Figure 3C).

The CIP + IBU TGA profile showed a multistep degradation event which occurred in the temperature range from 157 °C to 500 °C (Table 1). Like the TGA profiles of the pure compounds, the profile showed no sign of decomposition from room temperature to 180 °C. Arriving at this temperature, the first decomposition occurred, which may be attributed to the first degradation of the IBU component. This degradation then followed multiple decomposition steps that occurred at around 270 °C, 295 °C, and 320 °C from 10 °C/min, 20 °C/min, 30 °C/min heating rate, respectively. Further degradation of the mixture occurred at the temperature of 305 °C, 330 °C, and 340 °C for 30 °C/min, 10 °C/min, and 20 °C/min, respectively. The final degradation of the mixture was observed at 460 °C, 480 °C, and 500 °C, corresponding to heating rates 20 °C/min, 10 °C/min, and 30 °C/min, respectively. In the DTA analysis of a CIP + IBU mixture, at (75 °C–100 °C) sharp endothermic peaks were observed corresponding to IBU melting point at differing heating rates (10 °C/min, 20 °C/min, and 30 °C/min). Broad endothermic peaks were observed typically around 200 °C–300 °C, indicating the melting of CIP, and decomposition of both drugs; these peaks grew with differing heating rates (30 °C/min > 20 °C/min > 10 °C/min). From 400 °C to 500 °C, two exothermic peaks were observed for the lower heating rate (10 °C/min and 20 °C/min), and endothermic peak was observed for 30 °C/min heating rate. These peaks indicate the final stages of the breakdown of organic matter, resulting in the formation of carbon dioxide (CO_2_) and other gaseous products. At lower heating rates, the system may have more time to complete oxidative or combustion reactions. Organic residues from CIP and IBU may undergo partial oxidation or breakdown reactions that release heat, causing exothermic peaks. At 30 °C/min, the faster heating does not allow sufficient time for exothermic decomposition processes to occur completely. Instead, endothermic processes may dominate because of faster decomposition.

### 3.2. Non-Isoconversional Kinetic Analysis

**Figure 5:**
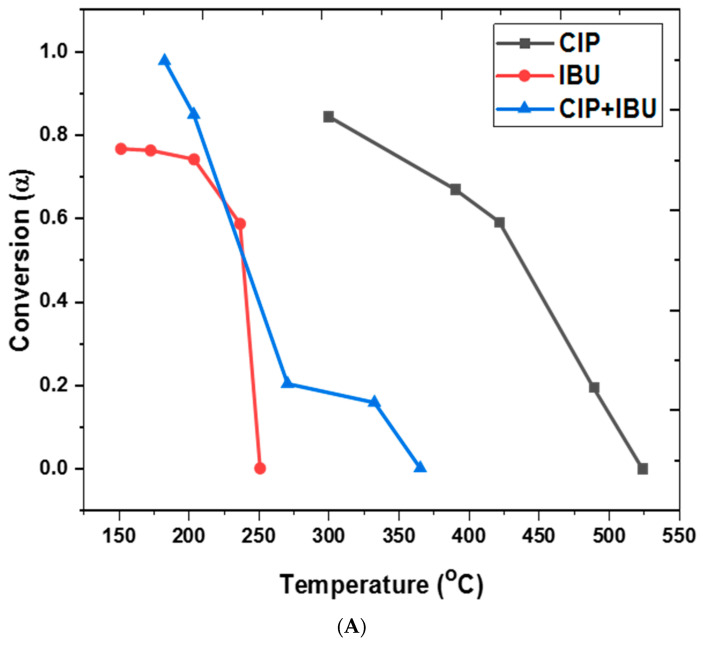

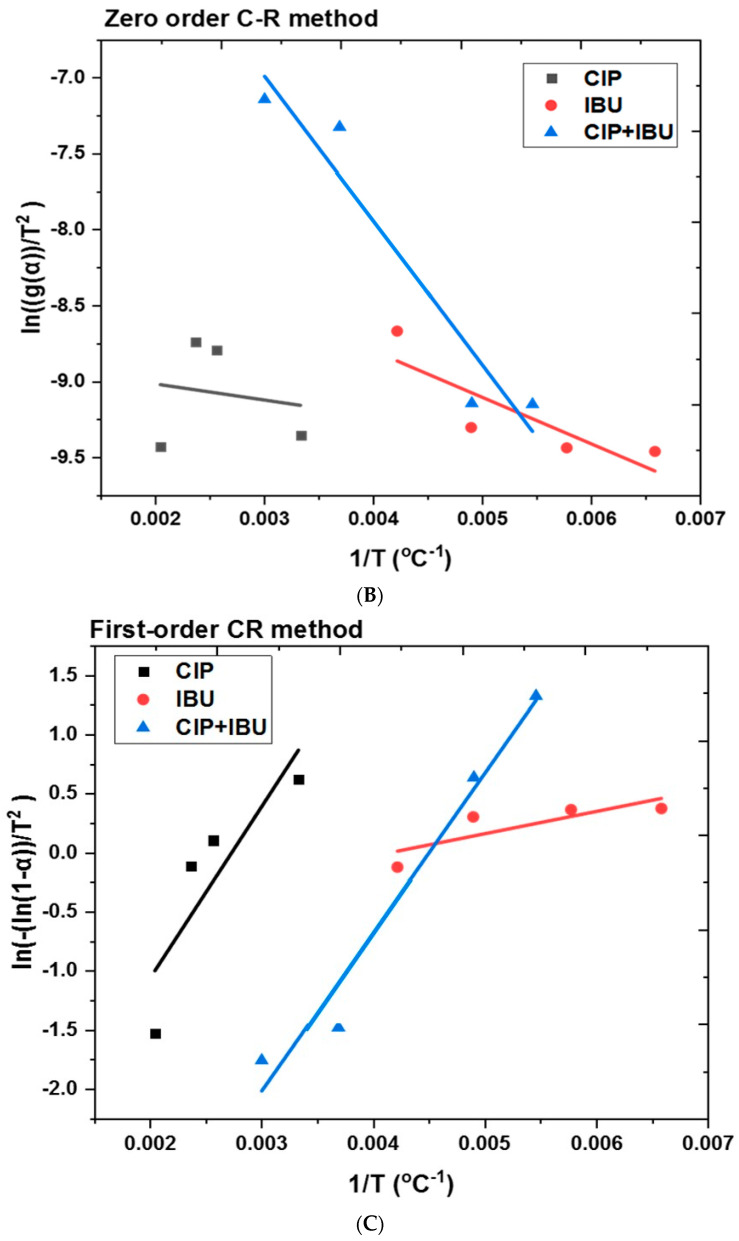
(**A**) Degradation conversion factors and Coats–Redfern kinetic modelling for (**B**) zero order (ZO) and (**C**) first order (FO), for the different pharmaceutical samples.

The non-isoconversional or model-fitting kinetic analysis was conducted using Coats–Redfern Kinetic model on the TGA data for the different samples. Two reaction orders were considered: zero order (ZO) and first order (FO). The model plot and summary of data obtained are presented in Figure 5 and Table 2. The table presents the activation energy (*E*), pre-exponential factors (*A*), and statistical indicators (R^2^ and RSSE) for ciprofloxacin (CIP), ibuprofen (IBU), and their mixture (CIP + IBU). The RSSE reflects the residuals between the observed and modeled data [3]. Lower RSSE values indicate better model accuracy. The first-order model shows lower RSSE for all cases. However, the highest error was recorded for the CIP FO model (0.6375) while the lowest was recorded for the IBU FO model (0.0552) (Figure 5 and Table 2).

Activation energy (*E*) signifies the energy threshold that reactants must overcome to initiate a chemical transformation [11]. *E* for CIP is significantly lower for the zero-order reaction (0.88 kJ/mol) compared to the first-order (12.07 kJ/mol), suggesting that the first-order model better captures the energy barrier for the decomposition of CIP. However, for IBU, the reverse was observed. The zero-order value was 2.54 kJ/mol, and the first-order is 1.57 kJ/mol, indicating that IBU’s degradation may occur more readily under the zero-order model. This can also be seen in the R^2^ values, which indicate how well the model fits the data. For CIP, the first-order model provides a much better fit (0.7481) compared to the zero-order model (0.0255) while for IBU, FO (0.6735) < ZO (0.7179) (Table 2). For CIP, the results strongly favor a first-order kinetic model. This aligns with previous studies on fluoroquinolone antibiotics, which often exhibit first-order degradation kinetics in aqueous environments. For instance, research by [32] on the degradation of CIP upon ozonation followed the first-order kinetics. The higher activation energy and better model fit for first-order kinetics in our results further support this trend. Interestingly, IBU shows a tendency towards zero-order kinetics when degrading alone. This agrees with earlier studies by [33]. However, Sabri et al. [34], who address photocatalytic degradation, typically report first-order kinetics for IBU breakdown.

Comparing both CIP and IBU activation energy (*E*) values, CIP was generally higher (Table 2). This difference provides information concerning the relative stability and reactivity of these two pharmaceuticals under thermal conditions. *E* represents the minimum energy required for a chemical reaction to occur. In the context of pharmaceutical degradation, higher *E* typically indicates that a compound is more stable and requires more energy input to initiate its breakdown. The fact that CIP shows higher *E* suggests it may be more resistant to degradation compared to IBU. This difference can be attributed to several factors including molecular structure of CIP. CIP has a more complex molecular structure compared to IBU, which is a relatively simpler nonsteroidal anti-inflammatory drug. The more complex structure of CIP may contribute to its higher stability [2] and thus higher *E* for degradation. The structure of CIP also includes bonds, particularly those in its aromatic rings and fluorine substituent [2], which are generally stronger than those in IBU [13]. Breaking these bonds requires more energy, which is reflected in the higher *E* values. For the environment, this finding suggests that CIP may persist longer in the environment compared to IBU. The persistence of antibiotics like CIP in the environment is a particular concern due to the potential for promoting antibiotic resistance in environmental bacteria [35].

For the mixture of ciprofloxacin and ibuprofen (CIP + IBU), the activation energies are 7.90 kJ/mol (ZO) and 11.18 kJ/mol (FO). This indicates a synergistic or competitive interaction between the two compounds, requiring higher energy for degradation compared to IBU alone, while CIP alone for the FO model slightly decreased (from 12.07 to 11.18 kJ/mol in CIP + IBU), suggesting an antagonistic interaction for CIP. Furthermore, for the mixture (CIP + IBU), the first-order model has the highest R^2^ (0.9676), meaning it fits the data almost perfectly, whereas the zero-order model (0.9249) also shows a good fit but is slightly less precise. The shift towards first-order kinetics for both drugs in the mixture, coupled with change in activation energies, suggests interactions that alter their degradation pathways. This phenomenon of altered kinetics in drug mixtures has been observed in other studies [36]. Ferreira [36] found that the presence of cellulose acetate affected the thermal degradation kinetics of IBU.

The pre-exponential factor (*A*) reflects the frequency of molecular collisions leading to reactions. These values reveal how easily the reactions are initiated, with lower *A* values indicating slower reaction rates and higher *A* values signifying faster reaction rates [11]. For CIP, the pre-exponential factor is much higher in the first order (5.24 min^−1^) than in the zero order (0.02 min^−1^), reinforcing the notion that a first-order kinetic model is more applicable. For IBU, the pre-exponential factor is also higher in the ZO model (4.74 min^−1^) compared to the FO model (0.84 min^−1^), suggesting a better model fit and more frequent reaction events under the zero-order assumption.

The CIP + IBU mixture shows an even more substantial difference between the zero-order (354.25 min^−1^) and first-order (360.68 min^−1^) models, indicating highly complex interactions between the two drugs that are more accurately represented by a first-order reaction model. The observed differences between individual and mixture kinetics suggests that pharmaceutical degradation in real-world scenarios can be more complex, where multiple compounds are often present simultaneously. This complexity is further highlighted by the near-perfect fit of the first-order model for the mixture, suggesting that the interaction between CIP and IBU creates a more predictable degradation pattern than either drug alone. This could be due to various factors, such as changes in the molecular environment, possible formation of intermediates, or alterations in the compounds’ reactivity when present together. Therefore, altered kinetics in mixtures suggest that degradation rates and pathways determined for individual compounds may not accurately predict their behavior in complex environmental matrices under thermal conditions.

### 3.3. Isoconversional Kinetic Analysis

**Figure 6:**
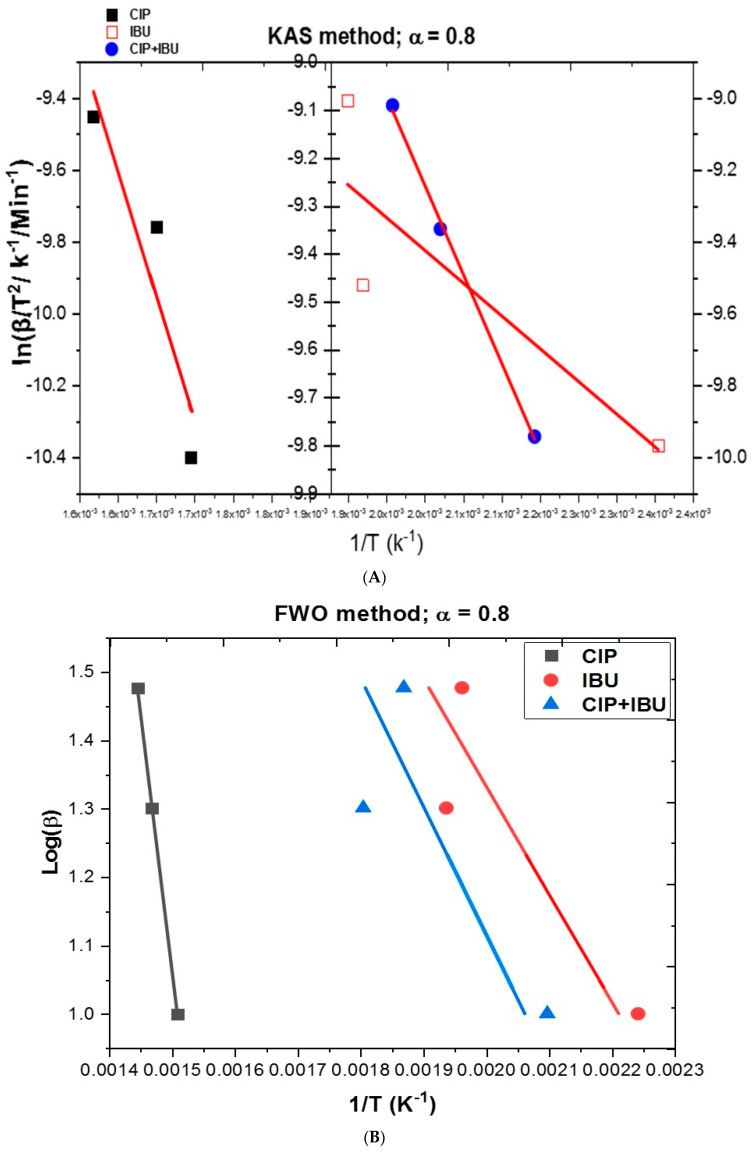

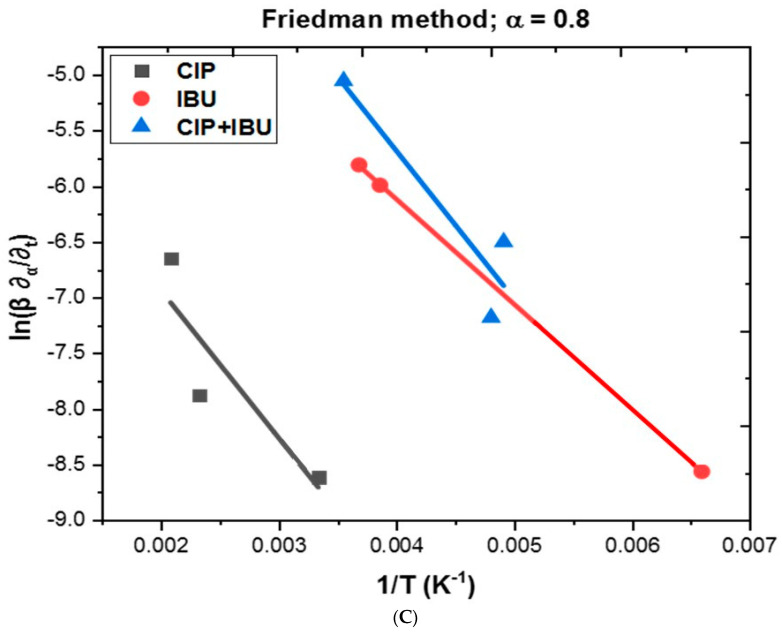
Various isoconversional kinetic model plots at degree of conversion (*α* = 0.8) for (**A**) KAS, (**B**) FWO, and (**C**) FR.

Isoconversional kinetic analysis is an essential method for investigating the degradation behavior of complex materials, providing a non-mechanistic approach to determining the kinetic parameters of a reaction [12,37]. Unlike model-fitting methods that rely on assuming a reaction mechanism, isoconversional models evaluate the kinetics directly from experimental data, offering flexibility and accuracy across different heating rates [12]. In this study, three well-established isoconversional methods were employed (Figure 6): Kissinger–Akahira–Sunose (KAS) (Figure 6A), Flynn–Wall–Ozawa (FWO) (Figure 6B), and FR models (Figure 6C). These methods provide valuable information concerning the degradation kinetics of emerging pharmaceutical pollutants, such as ciprofloxacin (CIP), ibuprofen (IBU), and their mixture (CIP + IBU) and the results are presented in Table 3. The data in Table 3 present the *E* (kJ/mol) and *A* (mins^−1^) derived from three isoconversional kinetic models at a degree of conversion of α = 0.8. Additionally, statistical parameters such as the coefficient of determination (R^2^) and residual sum of squares (RSSE) are included to assess the goodness of fit for each model.

The *E* value for CIP using the KAS model is 58.09 kJ/mol with a high pre-exponential factor (*A* = 47,578 min^−1^) (Table 3). The *E* value obtained suggests that CIP requires substantial energy for its thermal degradation, aligning with the literature. For instance, studies on the thermal decomposition of CIP facilitated by Co-AC catalysts have reported activation energy of 62.69 kJ/mol, computed by a linearized form of the Arrhenius equation [38]. Furthermore, a sludge-based and ruthenium/platinum catalyst-assisted thermal breakdown of CIP reported an *E* value of 53.8 kJ/mol [39] and 40 kJ/mol [40], respectively. The high pre-exponential factor indicates a fast degradation process, which reflects the molecular complexity of CIP and its potential for rapid breakdown once a critical energy threshold is reached. However, the relatively high RSSE (0.0620) in the KAS model suggests some deviation in the fitting, potentially due to variations in the degradation mechanisms at higher temperatures. In contrast, the FWO model shows a much lower *E* value for CIP at 2.42 kJ/mol, with a pre-exponential factor of 0.735 min^−1^ (Table 3). While this model yields an extremely high R^2^ value (0.9998), suggesting a near-perfect fit with the experimental data, the significantly lower *E* value implies that the FWO model captures a different aspect of the degradation process. This discrepancy between the KAS and FWO models could be attributed to the different assumptions made by each model. Furthermore, using the Friedman method, CIP shows an *E* value of 11.023 kJ/mol, with first-order (FO) and second-order (SO) *A* values of 0.069 min^−1^ and 0.343 min^−1^, respectively. This value is similar to the reported value of 16.2 kJ/mol for CIP thermo-catalytic breakdown by CuFe_2_O_4_@BC according to the Arrhenius equation based on the first-order kinetics at four different temperatures [41]. The Friedman method does not assume a specific reaction order but calculates the activation energy at different stages of the reaction [24]. The higher RSSE (0.421) and moderately high R^2^ (0.7866) indicate that this model captures the overall degradation kinetics but might miss some specific reaction stages.

The significant differences in *E* values for CIP between the KAS, FWO, and Friedman models indicate that CIP undergoes a complex degradation process involving multiple reaction steps. The KAS model, with its higher activation energy, likely captures the full complexity of the thermal degradation mechanism, including the dissociation of the quinolone ring structure, which requires substantial energy. The lower activation energies from the FWO and Friedman models may only represent the initial stages of degradation, such as side chain cleavage or other less energy-intensive processes. However, comparing all models for CIP based on R^2^ values, it followed that FWO > KAS > FR.

For IBU, the KAS model estimates an *E* value of 11.37 kJ/mol, with a relatively small pre-exponential factor (*A* = 1.887 min^−1^) (Table 3). This indicates that IBU requires significantly less energy for its degradation compared to CIP, as would be expected given the simpler molecular structure of IBU (Figure 1). The literature supports this lower energy requirement for IBU’s degradation [9]. The low *A* values further suggest that IBU undergoes a slower degradation process, which aligns with its stable molecular structure under moderate heating conditions [9]. Despite the lower *E* values, the R^2^ value of 0.7511, though moderate, still indicates a reasonably good fit between the experimental data and the model. The RSSE (0.0647) suggests a comparable level of deviation in the fit to CIP, indicating a similar level of accuracy for the KAS model in describing IBU degradation kinetics. In comparison, the FWO model yields a lower *E* values for IBU at 1.499 kJ/mol with an *A* value of 1.149 min^−1^, indicating a slower degradation rate (Table 2). The R^2^ value (0.8127) is relatively high, indicating a good fit, and the RSSE (1.07 × 10^−8^) is still quite low, suggesting an acceptable level of accuracy in predicting the degradation kinetics for IBU. Using the Friedman method, IBU shows an activation energy of 7.858 kJ/mol, which is higher than the FWO result but lower than the KAS model prediction. The first-order (FO) and second-order (SO) pre-exponential factors are 0.480 min^−1^ and 2.400 min^−1^, respectively (Table 2). These values indicate a more gradual degradation process at first (FO) followed by a more rapid breakdown in the second-order stage, which could suggest multiple degradation steps with different kinetics. The high R^2^ value (0.9999) obtained from the Friedman method suggests an almost perfect correlation between the experimental data and the model, with minimal deviation as indicated by the very low RSSE (9.85 × 10^−5^).

Across all models, the high R^2^ values, particularly for the Friedman method, demonstrate its strong accuracy in predicting IBU degradation kinetics (Table 3). This finding aligns with other studies where the Friedman method is favored for effectively handling multi-step reactions with high precision [13]. Additionally, the low RSSE values across all methods indicate that these models reliably capture the degradation profile of IBU, with the Friedman method performing exceptionally well. However, the activation energy (*E*) values for IBU obtained in our study significantly differ from those reported by Tita et al. [13] at the same degree of conversion (*α* = 0.8), where their values were 82.2 kJ/mol (Friedman), 95.2 kJ/mol (FWO), and 88.0 kJ/mol (KAS). Our study produced much lower activation energy estimates.

These differences can be largely attributed to the variation in heating rates between the studies. Tita et al. [13] used a range of lower heating rates (2.5, 5, 7.5, 10, and 15 °C/min), while our study applied higher rates of 10, 20, and 30 °C/min. Heating rates significantly influence the estimated *E* values by affecting the reaction rate and the temperature at which degradation occurs. Lower heating rates allow for more accurate determination of kinetic parameters, as they capture slower degradation steps with higher energy barriers [42]. Conversely, higher heating rates accelerate the degradation process, resulting in lower activation energy estimates because the reaction may proceed rapidly and bypass energy-intensive steps, often due to non-equilibrium conditions [42]. Therefore, the higher heating rates used in our study likely contributed to the lower activation energy values, capturing faster, less energy-demanding stages of degradation, while the lower rates in Tita et al.’s study allowed for a more comprehensive assessment of IBU’s full energy requirements. This difference in heating protocols explains the observed discrepancies in *E* values between the two studies.

Comparing the results between CIP and IBU samples, CIP generally shows higher activation energies than IBU across all models, indicating that CIP requires more energy to degrade thermally compared to IBU. This difference is consistent with their chemical structures. IBU shows much lower pre-exponential factors across all models, reflecting its lower energy barrier for degradation.

The results for the CIP and IBU mixture (CIP + IBU) show interesting trends in *E* values and model fitting, particularly when compared to the individual components (Table 3). For the KAS model, the *E* values for the mixture is 41.09 kJ/mol, with a relatively high pre-exponential factor (*A* = 12,050 min^−1^). The high R^2^ value of 0.9983 suggests excellent model performance, indicating that the kinetic behavior of the mixture is well captured by the model. The residual sum of squares error (RSSE) of 0.0007 is the lowest among all the samples, which implies a nearly perfect fit between the experimental data and the model. From the FWO model, the mixture *E* value (1.152 kJ/mol) is very similar to that of IBU singly (1.153 kJ/mol). This similarity suggests that IBU may dominate the thermal degradation process in the mixture, leading to comparable activation energies. The pre-exponential factor is also consistent with the FWO values for the individual components, reinforcing the idea that IBU plays a major role. However, the R^2^ value (0.6958) is slightly lower than that of CIP or IBU individually, which may indicate that the FWO model struggles to capture the exact interactions or combined effects within the mixture. Nonetheless, the RSSE (1.44 × 10^−8^) is still low, indicating that the model is reasonably accurate. For the Friedman model, the activation energy for the mixture is 11.080 kJ/mol, with pre-exponential factors of 3.505 min^−1^ (first-order) and 17.525 min^−1^ (second-order). The R^2^ value of 0.8568 is relatively high, suggesting that the model performs well in describing the mixture’s degradation kinetics. The RSSE (0.339) is comparable to that of CIP alone, implying that the degradation behavior of the mixture closely resembles that of the individual components.

Generally, The KAS model provides the best fit for the mixture (CIP + IBU) with an R^2^ of 0.9983 and an extremely low RSSE (0.0007), indicating that this model is particularly well-suited for capturing the kinetics of the combined system. Furthermore, the mixture shows intermediate activation energies compared to the individual components. This suggests that the interaction between the two compounds during degradation modifies the energy required, potentially due to synergistic (in case of IBU) or antagonistic effects (in case of CIP), implying that IBU may influence the overall degradation kinetics. The *A* value for the mixture is generally higher than for IBU but lower than for CIP alone. This pattern suggests that the presence of both compounds in the system affects the frequency of molecular collisions and the overall degradation rate.

### 3.4. Thermodynamics Analysis

**Figure 7:**
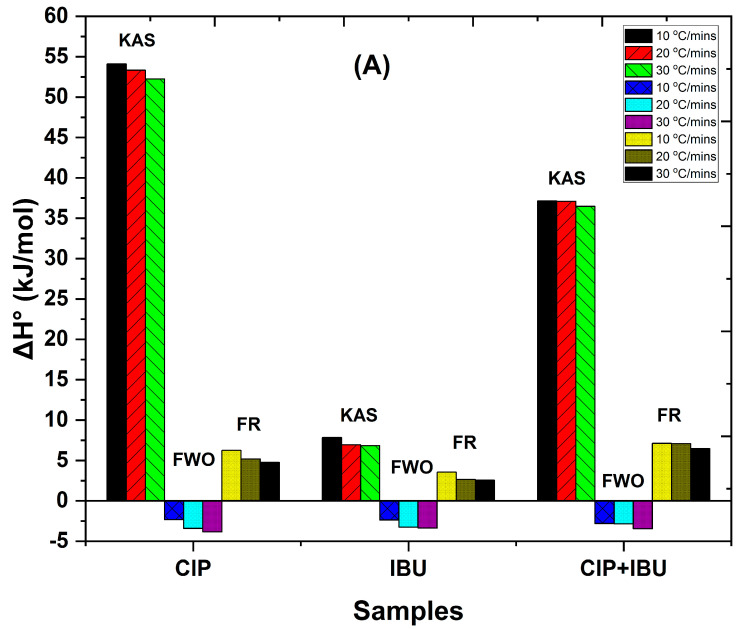

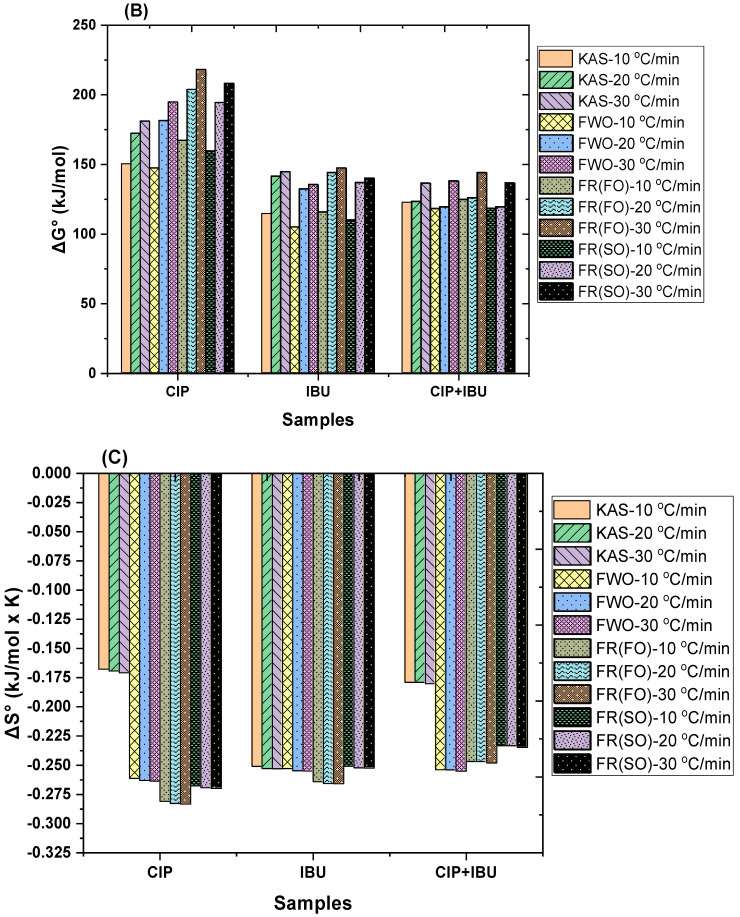
Thermodynamic characteristics showing (**A**) changes in enthalpy (∆*H*°, kJ/mol), (**B**) Gibbs free energy (∆*G*°, kJ/mol), and (**C**) entropy (Δ*S*°, kJ/mol × K) of thermal treatment at different heating rates for different pharmaceutical sample degradations for isoconversional or model-free kinetic models at degree of conversion (*α* = 0.8). KAS = Kissinger–Akahira–Sunose; FWO = Flynn–Wall–Ozawa; FR (FO) = Friedman First Order; and FR (SO) = Friedman Second Order.

Figure 7A–C displays the thermodynamic parameters, including entropy (Δ*S*°), Gibbs free energy (Δ*G*°), and enthalpy (Δ*H*°). The pre-exponential factor and activation energy values obtained using the model-free technique were used to study these parameters. These parameters were studied at the temperature of the conversion factor (*α* = 0.8). These temperatures were as follows: 300, 430, and 481.13 °C for CIP; 151.71, 259.28, and 271.83 °C for IBU; 203.74, 208.25, and 281.7 °C for CIP + IBU for heating rates of 10 °C/min, 20 °C/min, and 30 °C/min, respectively.

The amount of energy needed or used by a certain material to transform into another product is known as its enthalpy change. It is the distinction between the reactant and product enthalpies. An endothermic reaction is one in which the energy of the reactants is less than that of the products; this is shown by a positive Δ*H*° [11]. In this case, for the KAS method, all samples show positive Δ*H*° values, indicating endothermic processes. CIP (52.25 kJ/mol to 54.09 kJ/mol) has the highest values, followed by the CIP + IBU mixture (36.48 kJ/mol to 37.13 kJ/mol), then IBU (6.84 kJ/mol to 7.83 kJ/mol) (Figure 7A), indicating that the degradation process of CIP requires energy input. However, the FWO method shows negative Δ*H*° values for all samples (CIP: −2.34 to −3.84 kJ/mol > CIP + IBU: −2.8 to −3.46 kJ/mol > IBU: −2.38 to −3.38 kJ/mol), suggesting exothermic processes. This contradicts the KAS and FR results, which is interesting and may warrant further investigation. Generally, Δ*H*° values for the mixture are intermediate between those of CIP and IBU, suggesting that the degradation process reflects contributions from both pharmaceuticals. Furthermore, at higher heating rates (20 °C/min and 30 °C/min) (Figure 7A), there is a gradual decrease in Δ*H*°, suggesting that the degradation process becomes slightly less energy-intensive with increasing temperature. This implies that faster heating results in more efficient energy consumption for degradation. This could be due to the sample reaching the activation threshold more quickly.

The probability of a certain reaction happening is represented by the Gibbs free energy (Δ*G*°). A reaction that is not spontaneous is indicated by a positive value of Δ*G*°. As illustrated in Figure 7B, all Δ*G*° values are positive across all methods and heating rates, indicating non-spontaneous processes. When comparing the results from different kinetic models, the KAS model shows that CIP has the highest average Δ*G*° values (167.87 ± 0.93 kJ/mol), indicating that its degradation is the most thermodynamically unfavorable. IBU follows with an average Δ*G*° of 133.51 ± 0.55 kJ/mol, while the CIP + IBU mixture has a lower Δ*G*° of 127.51 ± 0.55 kJ/mol, suggesting slightly more favorable degradation conditions for the mixture compared to CIP alone. In the FR (First Order) model, CIP also exhibits the highest average Δ*G*° (196.44 ± 26.32 kJ/mol), reinforcing its status as the most energy-demanding compound for degradation. IBU has a lower Δ*G*° of 135.70 ± 17.39 kJ/mol, and the CIP + IBU mixture shows an average Δ*G*° of 131.53 ± 10.83 kJ/mol. This trend is consistent with the KAS model, where the mixture displays more favorable degradation properties than CIP alone. The FR (Second Order) model follows the same pattern, with CIP having an average Δ*G*° of 187.41 ± 25.08 kJ/mol, IBU at 129.00 ± 16.51 kJ/mol, and the CIP + IBU mixture at 124.78 ± 10.24 kJ/mol. These results confirm that CIP consistently requires the most external energy for degradation, while the mixture offers a more favorable, though still non-spontaneous, degradation pathway. Interestingly, the FWO model shows a slightly different trend. Here, CIP remains the most non-spontaneous with an average Δ*G*° of 174.52 ± 24.48 kJ/mol. However, the CIP + IBU mixture has an average Δ*G*° of 125.16 ± 11.13 kJ/mol, closely followed by IBU at 124.25 ± 16.76 kJ/mol. In this case, the difference between IBU and the mixture is minimal, suggesting that under the FWO model, the degradation processes of IBU and the mixture are almost equally thermodynamically unfavorable. Generally, the positive Δ*G*° values across all kinetic models and heating rates confirm that the degradation of CIP, IBU, and their mixture is non-spontaneous and requires external energy input. CIP consistently shows the highest Δ*G*° values, indicating that it has the most thermodynamically unfavorable degradation process, while the CIP + IBU mixture presents slightly more favorable conditions compared to CIP alone.

The results also showed that Δ*G*° values tend to increase with increasing heating rate for all kinetic models (Figure 7B). As the heating rate increases, the temperature of the reaction mixture rises more quickly, potentially leading to a more rapid decomposition. This rapid increase in temperature at higher heating rates may require more energy input to reach the activation state, which can manifest as higher Δ*G*° values. Essentially, the system may need to overcome greater energy barriers when the temperature rises swiftly, indicating that the process becomes less thermodynamically favorable at higher heating rates. This observation aligns with the principle that faster heating rates can lead to less time for the system to achieve equilibrium, thus requiring more energy to drive the reaction forward under non-equilibrium conditions [43].

Entropy (Δ*S*°) is a key thermodynamic parameter that measures the degree of disorder or randomness within a system. In the context of chemical reactions, the value of Δ*S*° provides insights into the reactivity of the substances involved. A more negative Δ*S*° typically signifies a greater reduction in disorder during the reaction, reflecting less molecular freedom in the final state compared to the initial state. Larger negative values can also imply reduced reactivity because the system becomes more ordered, limiting the ease with which molecules can interact or rearrange. In this study, all Δ*S*° values are negative across the different methods and heating rates (Figure 7C), suggesting that the degradation processes of the pharmaceutical compounds involve a transition state with a more ordered structure compared to the initial molecular state. This ordering likely reflects the formation of an organized transition complex during decomposition, even though the overall process eventually results in disordered gaseous by-products. This tendency toward an organized transition state could influence the thermal stability of these pharmaceutical compounds and their behavior in various environmental conditions. Among the compounds studied, IBU consistently exhibits the most negative Δ*S*° values, ranging from −0.25 ± 0.001 kJ/mol × K to −0.26 ± 0.001 kJ/mol × K. This suggests that IBU undergoes a significant decrease in entropy during the process, implying that its molecular arrangement becomes more ordered as the reaction proceeds, potentially due to stronger interactions or more constrained molecular configurations. The mixture of CIP + IBU shows intermediate Δ*S*° values, ranging from −0.18 ± 0.001 kJ/mol × K to −0.25 ± 0.001 kJ/mol × K, which suggests a moderate decrease in disorder compared to the individual components. This could indicate that the combined system has more molecular flexibility than IBU alone but still experiences a notable reduction in entropy during the degradation process. CIP exhibits the least negative Δ*S*° values, ranging from −0.17 ± 0.002 kJ/mol × K to −0.28 ± 0.001 kJ/mol × K. Although CIP shows some variability across the different methods and heating rates, its overall trend indicates a lesser reduction in molecular disorder compared to IBU, which could reflect differences in molecular structure, size, or interaction dynamics during degradation.

The differences in Δ*S*° values between the various methods (KAS, FWO, FR) and heating rates are relatively minor when compared to the larger variations observed in enthalpy (Δ*H*°) and Gibbs free energy (Δ*G*°). This suggests that while the overall level of disorder changes during the process, these changes are not as sensitive to the experimental conditions as the energetic parameters are. Nevertheless, the consistent negative Δ*S*° values reinforce the idea that these reactions proceed with a decrease in entropy, pointing to a more ordered final state in the degradation pathway of the pharmaceuticals.

## 4. Conclusions

This comprehensive thermoanalytical and kinetic study provides key insights into the thermal stability and degradation behavior of ciprofloxacin (CIP) and ibuprofen (IBU), two significant pharmaceutical pollutants. The findings reveal that CIP exhibits higher thermal stability and activation energy than IBU, consistent with its more complex molecular structure. The CIP + IBU mixture displays intermediate thermal properties, indicating possible synergistic or antagonistic interactions during degradation. Distinct degradation patterns were observed, with the Kissinger–Akahira–Sunose (KAS) model yielding the most consistent results for predicting kinetic behavior across the compounds. Heating rates influenced the degradation processes, with higher rates generally leading to less thermodynamically favorable conditions. Thermodynamic analysis shows that increasing the heating rate lowers the activation enthalpy (Δ*H*°) and makes the process slightly more favorable in terms of Gibbs free energy (Δ*G*°), especially for CIP. Entropy changes (Δ*S*°) indicate that degradation results in more ordered systems, with higher heating rates causing less pronounced reductions in disorder. The balanced thermodynamic profile of the CIP + IBU mixture highlights the impact of combined pharmaceutical compounds on degradation behavior. These findings enhance our understanding of the environmental fate of these pollutants and inform strategies for mitigating their ecological and health impacts. For effective thermal treatment, incineration systems should be optimized to operate above 550 °C for complete CIP breakdown and above 350 °C for IBU. Additionally, given the complex, multistep degradation observed in the CIP + IBU mixture, further research is needed to study other pharmaceutical mixtures to fully understand potential synergistic effects and improve wastewater-treatment processes.

## Figures and Tables

**Table 1:** Thermal properties of CIP, IBU, and CIP + IBU obtained from TGA curves.

Samples	Heating Rate (°C/ min)	T_onset_ (°C)	T_endset_ (°C)
CIP	10	300	550
	20	310	550
	30	320	550
IBU	10	152	350
	20	183	350
	30	190	350
CIP + IBU	10	157	500
	20	178	500
	30	185	500

**Table 2:** Activation energy and pre-exponential factors from Coats–Redfern model for the different pharmaceutical samples.

Parameters	CIP		IBU		CIP + IBU	
	ZO	FO	ZO	FO	ZO	FO
E (kJ/mol)	0.88	12.07	2.54	1.57	7.90	11.18
A (min^−1^)	0.02	5.24	4.74	0.84	354.25	360.68
R^2^	0.0255	0.7481	0.7179	0.6735	0.9249	0.9676
RSSE	0.3853	0.6375	0.1177	0.0552	0.2766	0.2266

ZO: zero order; FO: first order.

**Table 3:** Activation energy (*E*) and pre-exponential factor (*A*) derived using various isoconversional kinetic methods at conversion (*α*) = 0.8.

Samples	KAS				FWO				FR				
	*E* (kJ/mol)	*A* (min^−1^)	R^2^	RSSE	*E* (kJ/mol)	*A* (min^−1^)	R^2^	RSSE	*E* (kJ/mol)	*A* (min^−1^)		R^2^	RSSE
										First Order (FO)	Second Order (SO)		
CIP	58.09	47,578	0.8673	0.0620	2.426	0.735	0.9998	2.91 × 10^−13^	11.023	0.069	0.343	0.7866	0.421
IBU	11.37	1.887	0.7511	0.0647	1.153	1.499	0.8127	1.07 × 10^−8^	7.858	0.480	2.400	0.9999	9.86 × 10^−5^
CIP + IBU	41.09	12,050	0.9983	0.0007	1.152	1.500	0.6958	1.44 × 10^−8^	11.080	3.505	17.525	0.8568	0.339

## Data Availability

The original contributions presented in the study are included in the article, further inquiries can be directed to the corresponding authors.
